# Modulation of lncRNA H19 enhances resveratrol‐inhibited cancer cell proliferation and migration by regulating endoplasmic reticulum stress

**DOI:** 10.1111/jcmm.17242

**Published:** 2022-02-14

**Authors:** Tianye Li, Xinyue Zhang, Linglin Cheng, Chunting Li, Zihan Wu, Yingqi Luo, Kunpeng Zhou, Yanlin Li, Qi Zhao, Yongye Huang

**Affiliations:** ^1^ College of Life and Health Sciences Northeastern University Shenyang China; ^2^ School of Computer Science and Software Engineering University of Science and Technology Liaoning Anshan China

**Keywords:** apoptosis, cancer, ER stress, lncRNA, resveratrol

## Abstract

The phytoalexin resveratrol exhibits anti‐tumour activity in many types of cancer. In this study, we showed that resveratrol suppressed the survival of gastric tumour cells both in vivo and in vitro. Resveratrol promoted apoptosis, autophagy and endoplasmic reticulum (ER) stress in a dose‐dependent manner. RNA‐seq analysis showed that multiple cell death signalling pathways were activated after resveratrol treatment, while the use of ER stress activators (tunicamycin and thapsigargin) in combinatorial with resveratrol led to further inhibition of cancer cell survival. Results also showed that resveratrol altered the expression of several long non‐coding RNAs (lncRNAs), including MEG3, PTTG3P, GAS5, BISPR, MALAT1 and H19. Knockdown of H19 in resveratrol‐treated cells further enhanced the effects of resveratrol on apoptosis, ER stress and cell cycle S‐phase arrest. Furthermore, the migratory ability of resveratrol‐treated cells was dramatically decreased after H19 knockdown. In conclusion, resveratrol inhibited cancer cell survival, while knockdown of lncRNA H19 resulted in increased sensitivity to resveratrol therapy.

## 1 INTRODUCTION

In recent decades, cancer‐associated deaths have become the leading cause of human mortality. Among them, gastric cancer is the fourth leading cause of cancer deaths worldwide, with an estimated 0.77 million deaths in 2020.^1^ Chemotherapy is a common strategy for cancer treatment, and there have been numerous endeavours to design, develop, modify and evaluate anti‐cancer drugs. Natural compounds present a huge potential source of anti‐cancer drugs. Resveratrol, the best‐known polyphenolic stilbenoid, is abundant in grape skin and seeds^2^, ^3^ and exhibits many health beneficial properties, including anti‐oxidation and anti‐inflammatory activity. To date, a number of reports have documented the application of resveratrol in cancer treatment.^4^, ^5^ Resveratrol has been shown to inhibit cellular proliferation, progression and invasiveness in various types of cancers.^4^ Several clinical trials evaluating the effects of resveratrol in cancer patients have been performed. However, reports have identified a number of limiting factors for resveratrol in human studies, including low bioavailability and potential side effects, such as mild gastrointestinal discomfort.^6^ Therefore, exploration of the underlying mechanisms in order to discover more potent cancer therapy strategies based on resveratrol treatment is still in progress.

Cancer initiation and poor prognosis are often accompanied by abnormal induction of cell death. Therefore, a common treatment strategy for cancer therapy is the targeting of cell death mechanisms^7^; increasingly, studies of cell death induction in cancer have highlighted a role for chemotherapy in cancer treatment. Apoptosis, autophagic cell death and necroptosis are common programmed cell death mechanisms that play crucial roles in cancer development, progression and metastasis.^7^, ^8^, ^9^ Deciphering crosstalk among these cell death mechanisms will be helpful when considering the application of natural compounds in cancer treatment. The regulation of factors related to cell death induction has been shown to enhance the effect of natural compounds on inhibition of cancer cell survival.^10^, ^11^, ^12^, ^13^ Meanwhile, treatment with natural compounds frequently activates various signalling pathways, including endoplasmic reticulum (ER) stress. ER stress could either help to recover cellular homeostasis and maintain cell survival or it can induce cell death.^14^ In fact, both autophagy and ER stress have been found to contribute to the establishment of drug resistance in cancers.^15^, ^16^ Correct modulating of autophagy and ER stress could facilitate the anti‐tumour effects of natural compounds and help to overcome any potential subsequent chemoresistance.

Among the large numbers of RNA transcripts, protein‐coding sequences account for only a small fraction,^17^ and the remaining transcripts are non‐coding RNAs except for some that produce functional small peptides.^18^ Long non‐coding RNAs (lncRNAs) >200 nt in length modulate cell proliferation, cell death, differentiation, and metastasis via complex gene expression profiles and multiple mechanisms.^18^, ^19^ Regulation of the expression of lncRNAs may enhance the anti‐cancer function of natural compounds. For example, regulation of lncRNA MALAT1 plays a role in resveratrol‐inhibited colorectal cancer cell invasion and metastasis via the Wnt/β‐catenin signalling pathway.^20^ The lncRNA NEAT1 has also been shown to play a role in resveratrol‐mediated inhibition of proliferation, migration and invasiveness in multiple myeloma cells through modulation of the Wnt/beta‐catenin signalling pathway.^21^ However, reports documenting the investigation of lncRNA in resveratrol treatment are still limited. Therefore, the present study aimed to provide evidence in support of the application of resveratrol in gastric cancer treatment by examining the modulation lncRNA‐mediated cell death mechanisms.

## 2 MATERIALS AND METHODS

### 2.1 Cell proliferation assay

Cell counting kit‐8 (CCK‐8) (Bimake, Shanghai, China) assays were performed to investigate the effects of resveratrol treatment on cell proliferation. Cells were seeded in 96‐well plates at a density of 2 × 10^3^ cells per well and exposed to various concentrations of resveratrol for 24, 48, 72 and 96 h after cell adherence under normal cell culture conditions. CCK‐8 solution was then added to each well and incubated for an additional 1.5 h. Cell viability was analysed using a microplate reader at 450 nm.

### 2.2 Subcutaneous tumorigenesis in nude mice

The animals were cared for in accordance with the Guide for the care and use of laboratory animals in China. All experimental procedures were approved by the Animal Care and Use Committee of the Northeastern University Committee, China.

Four‐week‐old male BALB/c nude mice were procured from Changsheng Biotechnology (Liaoning, China). The mice were grouped‐housed under specific pathogen‐free conditions, at a temperature of 24°C with a relative humidity of 50%–60%, under a 12‐h‐light/12‐h‐dark schedule. Animals were provided ad libitum access to standard food and drinking water. All mice were healthy and infection‐free during the experimental period. All surgical procedures were performed under aseptic conditions. SGC7901 cells (3 × 10^7^ cells/ml) were injected into the right super lateral subcutaneous tissue of the nude mice. Tumour growth was measured with callipers every 3 days, and tumour volume was calculated according to the following equation: tumour volume (mm^3^) =0.5 × the longest diameter ×the shortest diameter^2^. When the mean tumour volume reached approximately 80 mm^3^, mice were randomized into the vehicle control (100 μl physiological saline solution/2 days) and resveratrol‐treated (25 mg/kg/2 days) groups. The drug was administered by intraperitoneal injection. At the termination of the experiment, the mice were euthanized by cervical dislocation, and tumours were harvested and weighed.

### 2.3 Protein extraction and western blotting

For whole‐cell lysates, treated SGC7901 cells were washed with PBS and lysed in RIPA buffer (Beyotime, Hangzhou, China) containing 1 mM phenylmethanesulfonyl fluoride (PMSF) (Beyotime, Hangzhou, China). To obtain cytoplasmic and nuclear proteins, the Nuclear and Cytoplasmic Protein Extraction Kit (Beyotime, Hangzhou, China) was used according to the manufacturer's instructions. Protein extract concentrations were measured using the bicinchoninic acid (BCA) protein assay kit (Beyotime, Hangzhou, China). Equal amounts of protein (20 μg) were separated by 12% sodium dodecyl sulphate polyacrylamide gel electrophoresis (SDS‐PAGE), transferred to polyvinylidene difluoride (PVDF) membranes, blocked in 5% non‐fat milk solution for 1 h at room temperature and incubated with specific primary antibodies (Table S1) overnight at 4°C. The membranes were then incubated with the appropriate HRP‐conjugated secondary antibody at room temperature for 1 h, and visualized using an ECL detection system. To ensure equal loading, GAPDH or β‐actin served as whole‐cell reference genes. The reference genes for cytoplasm and nucleus were GAPDH and H3 respectively.

### 2.4 Soft agar cloning formation assay

A soft agar cloning formation assay was used to detect the survival ability of resveratrol‐treated SGC‐7901 cells. In brief, 6‐well plates were coated in 1.5 ml culture medium with 0.6% soft agar. After solidification, 1 × 10^3^ resveratrol‐treated cells in 1 ml culture medium with 0.35% soft agar were added to each well of the coated plates. After colony formation for 20 days in a 37°C incubator, the plates were washed with PBS, fixed with 4% paraformaldehyde and stained with 0.1% crystal violet. The stained colonies were photographed and counted for further analysis. There were three replicates for each treated group.

### 2.5 Immunofluorescent detection

Immunofluorescent assays were used to analyse β‐catenin localization and expression. Treated SGC7901 cells were seeded into a 24‐well plate at 5 × 10^4^ cells per well and fixed with 4% paraformaldehyde for 30 min, permeabilized with 0.2% Triton X‐100 for 30 min and blocked in 1% bovine serum albumin (BSA) in PBS for 30 min at room temperature. Incubation with β‐catenin primary antibody was then carried out at 4°C overnight. After a further washing step in PBS plus 0.2% Tween‐20, the cells were incubated overnight with the appropriate fluorescence‐conjugated secondary antibody at 4°C, in the dark. Nuclei were stained with Hoechst33342 for 5 min at room temperature. A fluorescence microscope was used to observe cells and collect images.

### 2.6 Migration assay

Transwell assays were used to determine cell migration ability. The assays were performed using a 24‐well transwell chamber (8 µm pore size). Briefly, 2 × 10^4^ resveratrol‐ and/or lncRNA H19 siRNA‐treated cells were suspended in 100 μl serum‐free culture medium and added into the upper chamber, and 600 μl medium containing 2.5% FBS was added to the lower chamber of each well. After incubating for 24 h, the chambers were washed with PBS, and cells were fixed with methanol and stained with 0.1% crystal violet. After staining, the chambers were washed with PBS. Five fields were randomly selected and observed under the microscope.

### 2.7 Statistical analysis

All experiments were repeated three times, and data were expressed as mean ± standard error of the mean (SEM). One‐way ANOVA was used to determine the significance of multiple comparisons, and the unpaired Student's *t*‐test was used to assess the differences between two groups. A *p*‐value < 0.05 was considered statistically significant.

## 3 RESULTS

### 3.1 Resveratrol affected cell survival by inhibiting proliferation and increasing apoptosis

**FIGURE 1:**
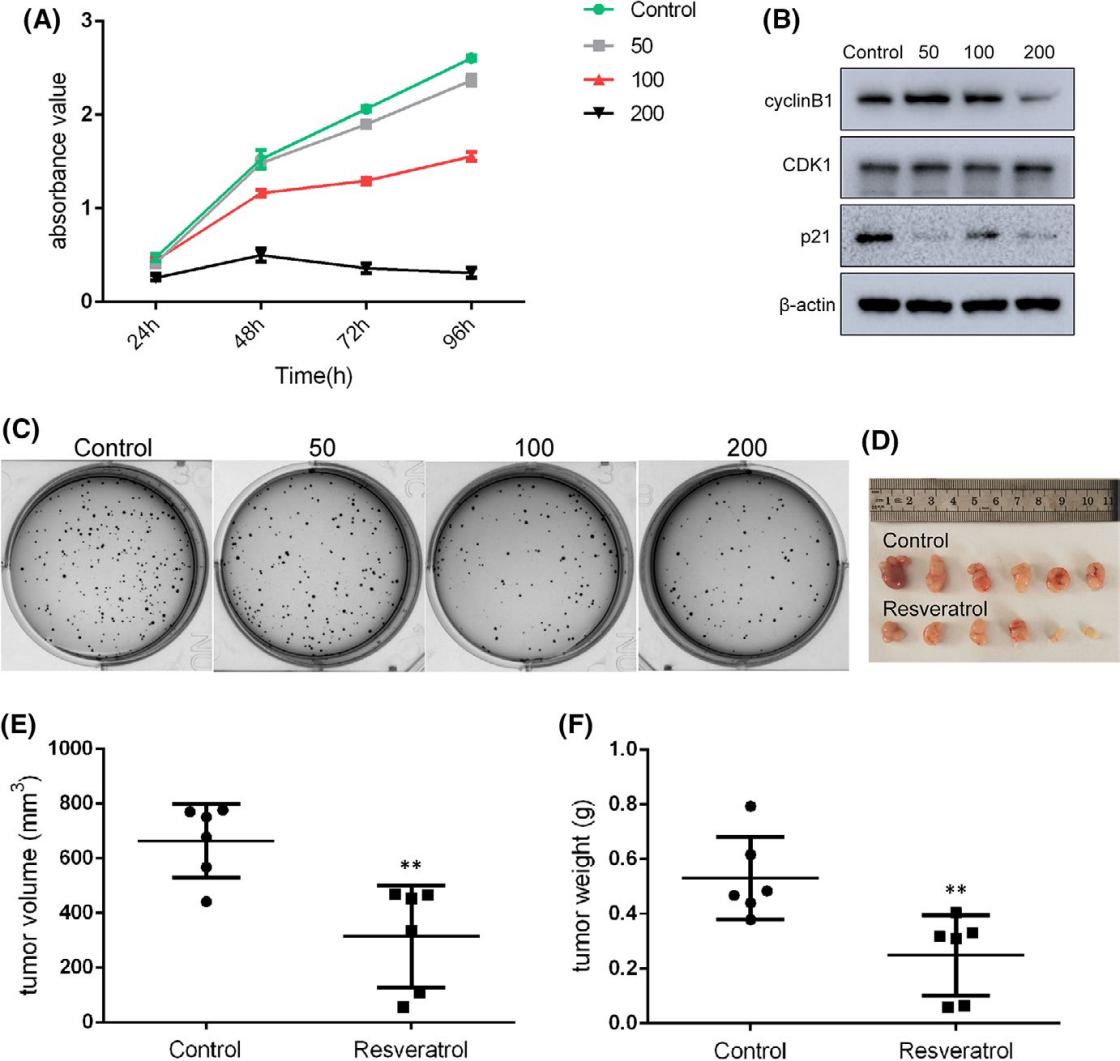
Resveratrol affected SGC7901 gastric cancer cells survival in vitro and in vivo. (A) Proliferation of cancer cells at different resveratrol concentrations and time periods of treatment. (B) Levels of cell cycle arrest‐associated proteins, as determined by Western blot analysis. (C) Colony formation in cells treated with 0, 50, 100 and 200 μM resveratrol. (D) SGC7901 cells were injected into BALB/c nude mice, as indicated. After therapy with 25 mg/kg/2d resveratrol for 20 days, mice were sacrificed in order to harvest the tumours. The tumour volume (E) and weight (F) were reduced by resveratrol treatment compared with the control group. Reported values are mean ± SEM. **p < 0.01 indicates significant differences compared with the control group

To determine the effects of resveratrol on gastric cancer cell viability in vitro, CCK‐8 assays were performed in order to analyse the effects of various concentrations resveratrol (0, 50, 100 and 200 µM) on the SGC7901 gastric cancer cell line over different time periods (24, 48, 72 and 96 h). The results suggested that resveratrol was able to inhibit cell proliferation in a dose‐ and time‐dependent manner, with significant inhibition of cell survival induced by 50 µM resveratrol after 24 h (Figure 1A). We chose this time period (24 h) for cell treatment with 0, 50, 100 and 200 µM resveratrol in the follow‐up study. Treatment with resveratrol significantly decreased the number of colonies formed, in a dose‐dependent manner (Figure 1C). Cell cycle arrest is always involved in the inhibition of proliferation, as shown in Figure 1B, levels of cyclin B1 were significantly downregulated by exposure to 200 µM resveratrol, while resveratrol also decreased the level of p21. The results therefore showed that resveratrol treatment induced cell cycle arrest. SGC7901 tumour‐bearing mice were then used to confirm the suppressive effect of resveratrol in vivo. Results showed that resveratrol treatment significantly reduced the tumour volume and weight compared with controls (Figure 1D–F).

**FIGURE 2:**
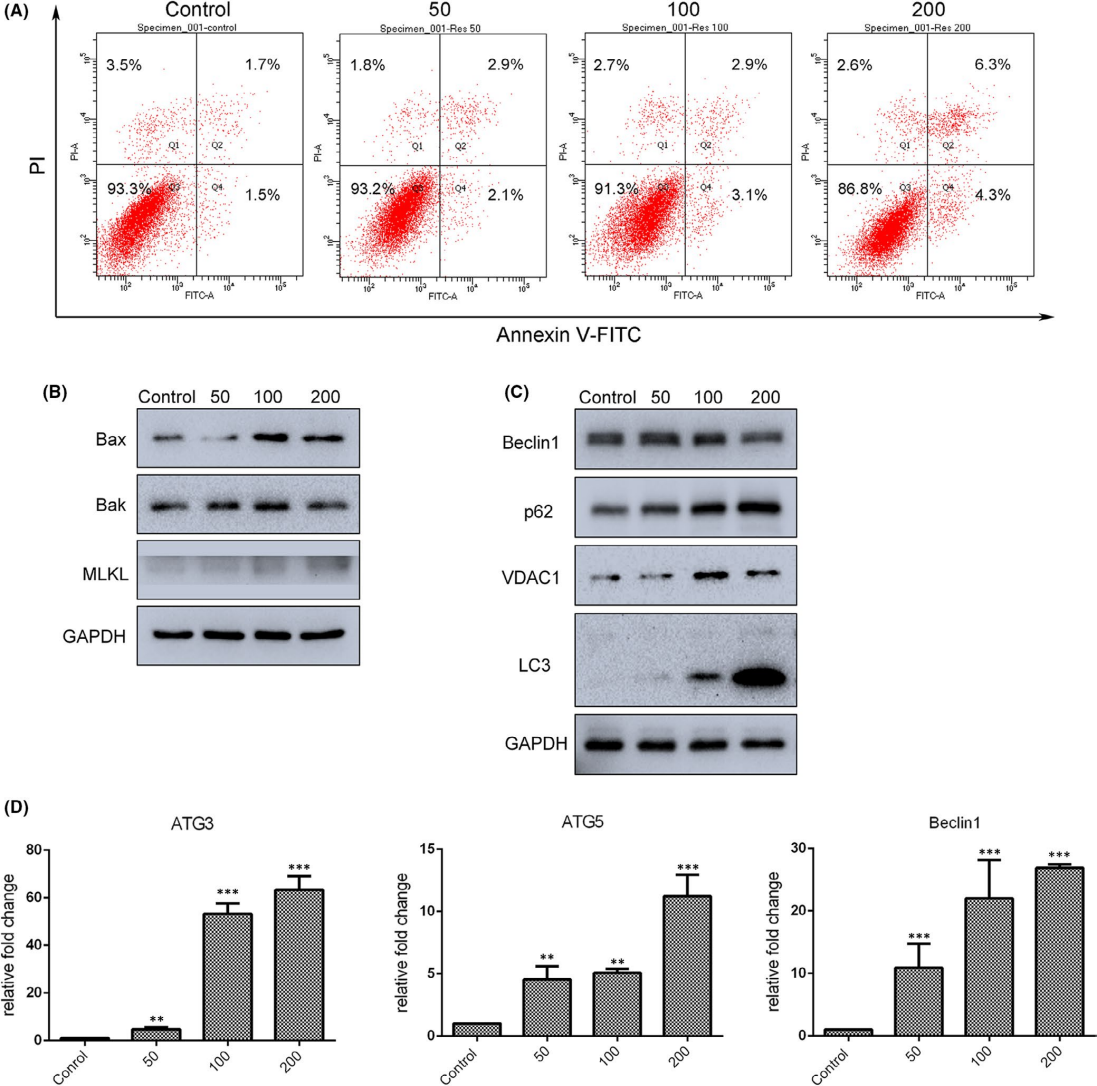
Apoptosis and autophagy were triggered by resveratrol in SGC7901 cells in dose‐dependent manner. (A) Annexin V/PI staining and flow cytometry were used to assess cellular apoptosis after treatment with different concentrations of resveratrol for 24 h. (B) Levels of apoptosis‐ and necrosis‐associated proteins, as determined by Western blot analysis. (C) Levels of autophagy‐related proteins, as determined by Western blot analysis. (D) Expression of autophagy associated genes in resveratrol‐treated cells was analysed by qRT‐PCR. Reported values are mean ± SEM. **p < 0.01 and ***p < 0.001 indicate significant differences compared with the control group

To further explore the roles of resveratrol in gastric cancer, detailed analyses of canonical cell death modes, including apoptosis, necrosis and autophagic cell death, were performed. As shown in Figure 2A, 200 µM resveratrol dramatically increased the rate of apoptosis of SGC7901 cells, as detected by flow cytometry and Annexin V‐FITC/PI staining. In addition, Western blot assay confirmed an increase in the levels of apoptosis‐related proteins; levels of Bax and Bak, as well as necrosis‐related protein MLKL, were enhanced after resveratrol treatment (Figure 2B). To confirm these findings, the effect of resveratrol on cell survival was examined in A549 lung cancer cells, as well as in another gastric cancer cell line, BGC823; CCK‐8 assays were used to detect the cell survival rate. The results indicated that resveratrol dramatically decreased the proliferation of A549 (Figure S1A) and BGC823 (Figure S1B) cells in a dose‐dependent manner, and significant downregulation of cellular proliferation was observed with 50 µM resveratrol. To identify the underlying mechanisms involved in the anti‐tumour effects of resveratrol, RNA‐seq analysis was performed in A549 lung cells treated with 0 and 50 µM resveratrol. Gene Ontology (GO) and Kyoto Encyclopedia of Genes and Genomes (KEGG) enrichment analysis were conducted in order to determine the role of differentially expressed mRNAs. As expected, we identified enhanced expression of a large group of genes related to apoptosis and autophagy (Figure S2A) and significant enrichment of pathways related to p53 and apoptosis (Figure S2B) in resveratrol‐treated cells. In addition, Gene Set Enrichment Analysis (GSEA) further confirmed that upregulated gene pathways were associated with p53 and Myc targets (Figure S3). In summary, cell survival was reduced by resveratrol treatment in a dose‐dependent manner.

### 3.2 Resveratrol enhanced autophagy by regulating multiple signalling pathways

Autophagic cell death is a distinct form of programmed cell death that plays an important role in maintaining cellular homeostasis. As shown in Figure 2C, the levels of autophagy‐related proteins p62 and LC3‐II and mitophagy‐related protein VDAC1 were upregulated after resveratrol treatment SGC7901 cells, in a dose‐dependent manner. ATG3, ATG5 and Beclin1 mRNA levels were also quantified by qRT‐PCR; the results demonstrated that expression of these genes was enhanced after 24‐h treatment with different concentrations (0, 50, 100 and 200 µM) of resveratrol (Figure 2D). However, as shown in Figure 2C, 200 µM resveratrol decreased the level of Beclin1 protein. These results indicate that resveratrol‐induced autophagy may be independent of Beclin1 expression.

**FIGURE 3:**
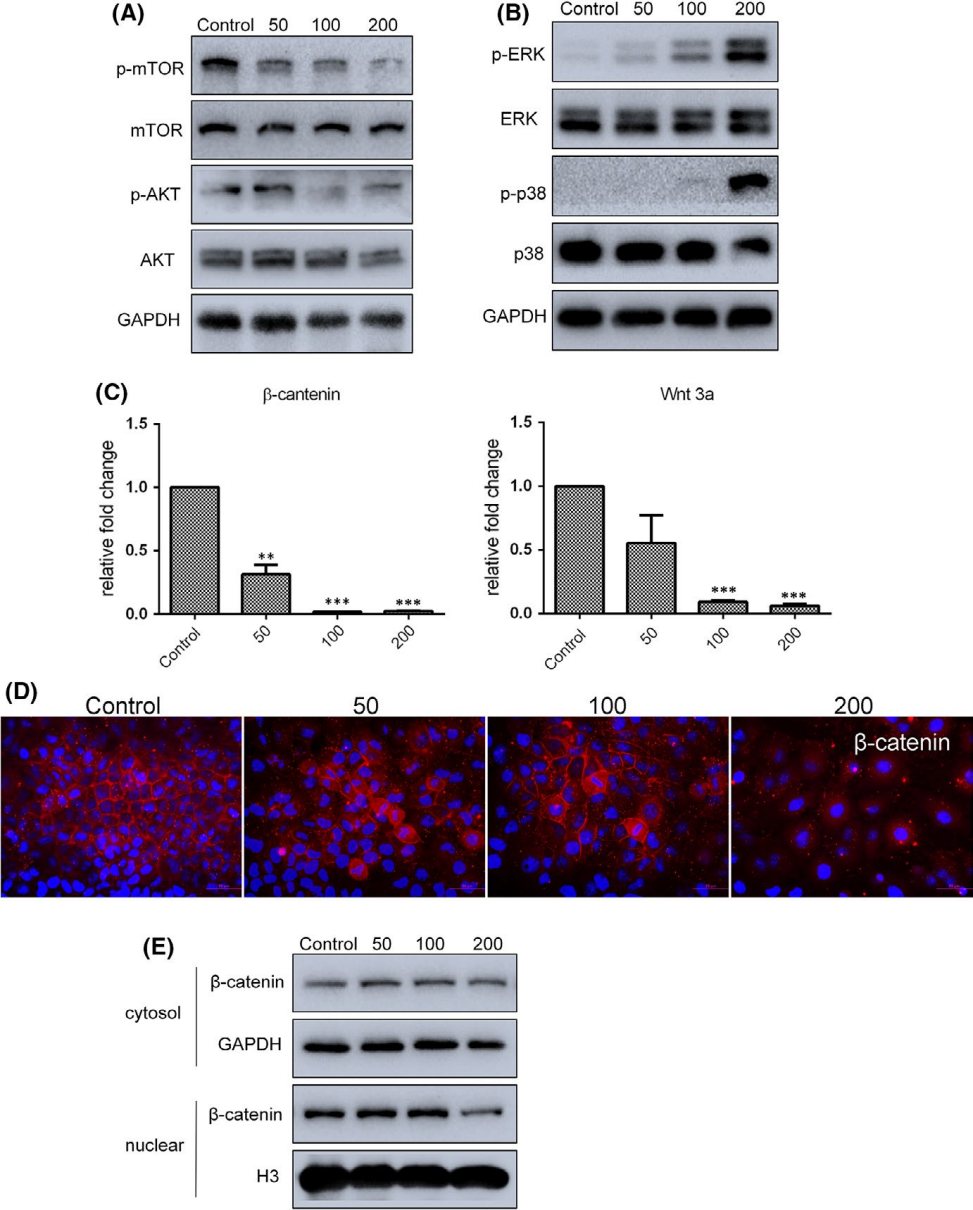
Several cell signalling pathways were analysed after resveratrol treatment. (A and B) Levels of AKT/mTOR and p38 MAPK/ERK pathway proteins, as determined by Western blot analysis. (C) mRNA expression of β‐catenin and Wnt3a determined by qRT‐PCR. (D) Distribution of β‐catenin (red) in resveratrol‐treated SGC7901 cells as determined by immunofluorescence. Nuclei were stained with Hoechst 33342 (blue). (E) Levels of nuclear β‐catenin, as evaluated by Western blot analysis. Reported values are mean ± SEM. **p < 0.01 and ***p < 0.001 indicate significant differences compared with the control group

The AKT/mTOR pathway is known to be essential for the regulation of autophagy. Analysis of AKT/mTOR phosphorylation by Western blotting indicated that exposure of SGC7901 cells to resveratrol for 24 h resulted in reduced levels of both phosphorylated AKT (Ser 473) protein and phosphorylated (activated) mTOR (Ser2448) (Figure 3A). ERK and Wnt/β‐catenin are important pathways for cell survival. As shown in Figure 3B, phosphorylation of p38 MAPK and ERK was found to be upregulated after treatment with 50, 100 and 200 µM resveratrol. The expression of both β‐catenin and Wnt3a mRNA was downregulated in resveratrol‐treated cells (Figure 3C), while β‐catenin was also decreased in nuclear protein extracts from cells treated with 200 µM resveratrol (Figure 3E). Meanwhile, immunofluorescence also confirmed that resveratrol treatment prevented the nuclear translocation of β‐catenin (Figure 3D) To summarize, these results indicate that Beclin‐1‐independent autophagy was triggered in SGC7901 cells exposed to resveratrol via enhanced LC3‐II formation and p62 accumulation, and AKT/mTOR, p38 MAPK/ERK and Wnt/β‐catenin pathways were involved in this process.

### 3.3 Endoplasmic reticulum stress enhanced resveratrol‐suppressed proliferation and migration of human gastric cancer cells

**FIGURE 4:**
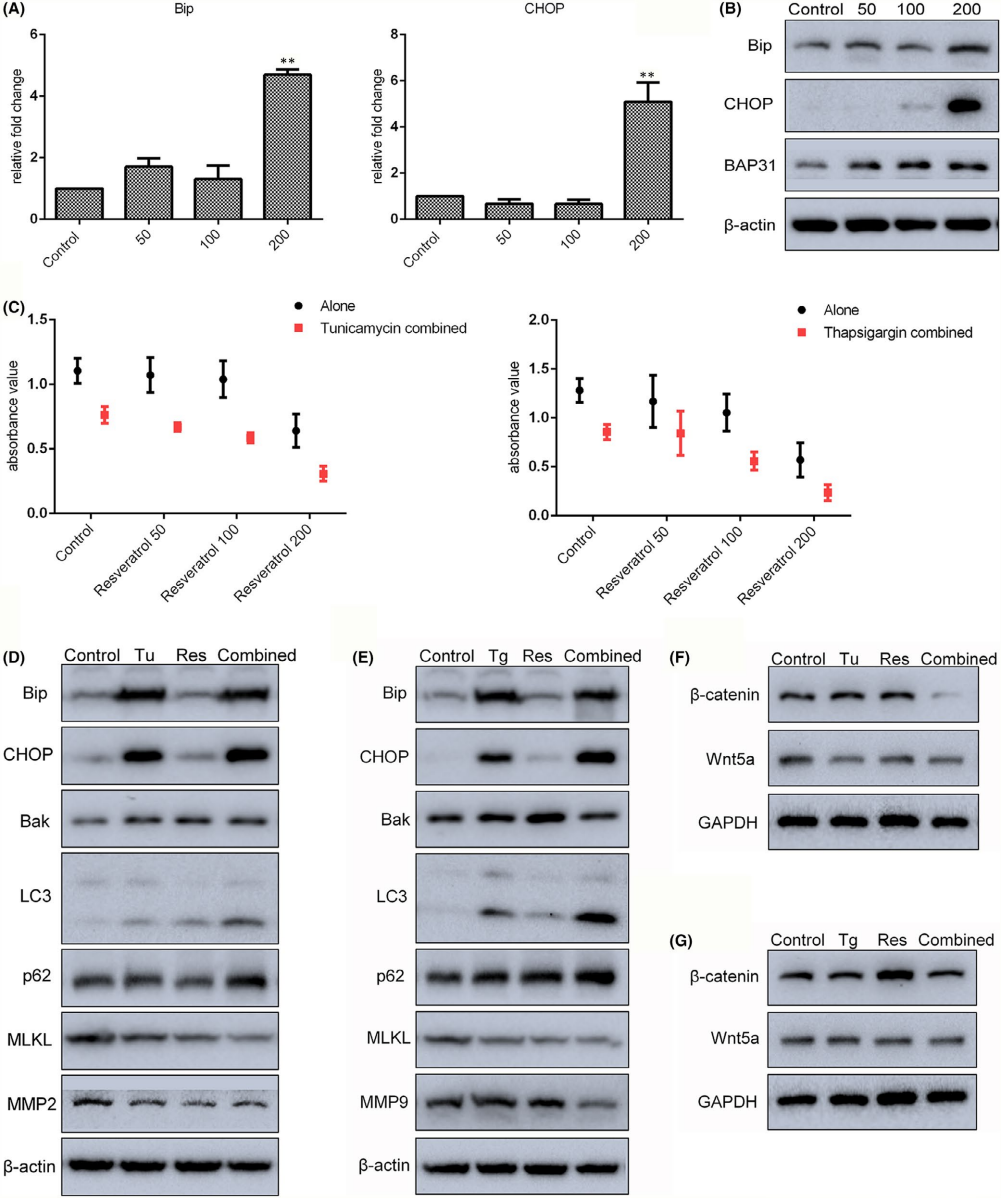
Endoplasmic reticulum stress cooperated with resveratrol (Res) at low concentration to increase gastric cancer cell death. (A and B) Expression of ER stress‐associated genes in resveratrol‐treated cells was analysed by qRT‐PCR and Western blot. (C) The survival rate of cells treated with 1 ng/ml tunicamycin (Tu) or 5 ng/ml thapsigargin (Tg) combined with 0, 50, 100 or 200 µM resveratrol (for 24 h) was detected by CCK‐8 assay. (D and E) Levels of ER stress‐, apoptosis‐, necrosis‐, autophagy‐ and migration‐related proteins in cells treated with combined resveratrol and tunicamycin/thapsigargin were analysed by Western blot. (F and G) Levels of β‐catenin and Wnt5a in combination‐treated cells were detected by Western blot. Reported values are mean ± SEM. **p < 0.01 indicates significant differences compared with the control group

Endoplasmic reticulum (ER) stress is a protective cellular stress response, persistent or intense ER stress can lead to programmed cell death or apoptosis. Bip, CHOP and BAP31 are all ER stress‐related proteins. The levels of both Bip and CHOP protein and mRNA were significantly increased with 200 µM resveratrol treatment; enhanced levels of BAP31 protein were detected after treating SGC7901 cells with resveratrol for 24 h (Figure 4A,B). These results indicate that resveratrol‐treated cells were undergoing ER stress.

Tunicamycin and thapsigargin induce ER stress by inhibiting the synthesis of glycoproteins and regulating calcium homeostasis respectively. In this study, SGC7901 cells were exposed to 1 ng/ml tunicamycin or 5 ng/ml thapsigargin combined with 0, 50, 100 or 200 µM resveratrol for 24 h, and the CCK‐8 assays were then used to determine the rate of cell survival. The results revealed that tunicamycin/thapsigargin in combination with resveratrol significantly reduced cell viability compared with either controls or cells treated with tunicamycin/thapsigargin or resveratrol alone (Figure 4C). Next, the levels of proteins related to ER stress, apoptosis, autophagy, necrosis and migration in tunicamycin/thapsigargin‐ and/or 50 µM resveratrol‐treated cells were determined. Consistent with the results of CCK‐8 assay, cells treated with tunicamycin/thapsigargin in combination with resveratrol exhibited increased levels of Bip, CHOP, Bak, LC3‐II, and p62 and decreased levels of migration‐related proteins MMP2 and MMP9 (Figure 4D,E). In addition, as shown in Figure 4F,G, the levels of both Wnt5a and β‐catenin were also decreased in cells with the combined treatment. These results suggest that ER stress may play a role in resveratrol‐induced inhibition of gastric tumour cell survival.

### 3.4 Resveratrol inhibited gastric cancer cell migration by reducing EMT‐associated genes expression

**FIGURE 5:**
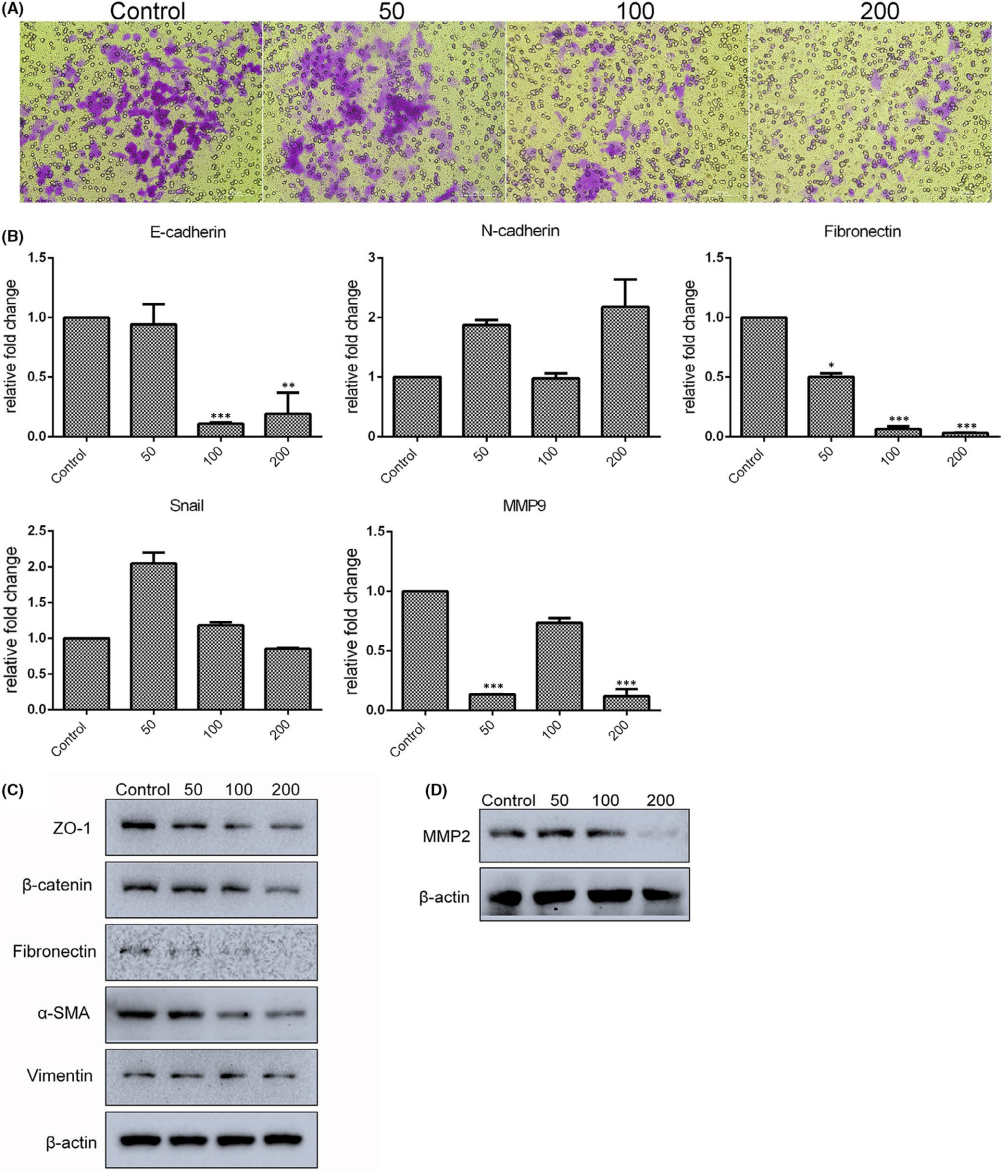
Migration ability of SGC7901 cancer cells was decreased with resveratrol treatment. (A) Migration of resveratrol‐treated cells was measured by transwell assays. Cells that had undergone migration were stained with 0.1% crystal violet. (B) mRNA expression of EMT‐associated genes was determined by qRT‐PCR. (C and D) Levels of EMT‐related proteins in resveratrol‐treated cells were detected by Western blot. Reported values are mean ± SEM. *p < 0.05, **p < 0.01 and ***p < 0.001 indicate significant differences compared with the control group

As major characteristics of cancer, invasiveness and metastatic ability are known to be closely associated with the establishment of drug resistance. The migratory ability of resveratrol‐treated cells was analysed by transwell assay; results showed that migration was dramatically decreased in a dose‐dependent manner after treatment with 0–200 µM resveratrol for 24 h (Figure 5A). To confirm these results, the expression of epithelial‐mesenchymal transition (EMT)‐associated genes was examined. Results of qRT‐PCR analysis revealed that expression of E‐cadherin, fibronectin and MMP9 was significantly decreased after treatment with 200 µM resveratrol for 24 h. Although expression of N‐cadherin and snail was increased after treatment with 50 µM resveratrol, the difference was not significant (Figure 5B). Cellular levels of ZO‐1, β‐catenin, fibronectin, α‐SMA, vimentin and MMP2 proteins were found to be downregulated by resveratrol (Figure 5C,D). These results suggest that resveratrol may inhibit cell migration by affecting EMT‐related gene expression.

### 3.5 Expression profile of LncRNAs in resveratrol‐treated cells

There is increasing evidence indicating that lncRNAs are critical for the initiation and progression of cancer. RNA‐seq analysis identified a total of 179 up‐ and 64 downregulated lncRNAs in 50 µM resveratrol‐treated A549 cells, compared with control group. LncRNA expression in the resveratrol group was significantly different from that in control group, as shown in the heat map (Figure S2C). Therefore, to further uncover the underlying mechanisms of action of resveratrol in cancer treatment, the present study examined several candidate lncRNAs that have demonstrated regulation in cancer progression. Results showed that the expression of lncRNAs MEG3, PTTG3P and BISPR was increased in resveratrol‐treated SGC7901 cells, and GAS5 expression was significantly decreased by 200 µM resveratrol treatment. However, resveratrol did not change the expression of lncRNAs DICER‐AS1, TUG1 or LINC01121. Interestingly, H19 and MALAT1 expression increased in cells treated with 50 µM resveratrol, while 200 µM resveratrol downregulated H19 expression (Figure S1C). We also used A549 and BGC823 cell lines to confirm the expression of H19 after treatment with 0–200 µM resveratrol for 24 h. As shown in Figure S1A, resveratrol‐treated A549 cells showed decreased viability and increased H19 expression, while BGC823 cells also exhibited a high level of H19 expression and similar reduced viability (Figure S1B). Therefore, we chose lncRNA H19 for further investigation of function in resveratrol‐treated gastric cancer cells.

### 3.6 LncRNA H19 knockdown enhanced sensitivity to resveratrol‐mediated inhibition of cell survival and migration

A specific H19 siRNA was applied to knock down the expression of H19. As shown in Figure S4, H19 expression was significantly knocked down through the use of H19 siRNA. Meanwhile, the expression of a number of other lncRNAs was investigated. For the majority, expression was not significantly affected by H19 knockdown, apart from the migration‐related lncRNA MALAT1, which decreased dramatically. This suggested that H19 may be involved in the process of cellular metastasis. To examine the impact of lncRNA H19 knockdown on the anti‐tumour effects of resveratrol treatment, 50 µM resveratrol was used in combination with H19 siRNA. The results demonstrated that the expression of anti‐tumour lncRNAs DICER‐AS1, TUG1, GAS5 and LINC01121 was significantly increased by 50 µM resveratrol in combination with H19 knockdown, while expression of MALAT1 in cells was significantly downregulated by the combined treatment (Figure S4).

**FIGURE 6:**
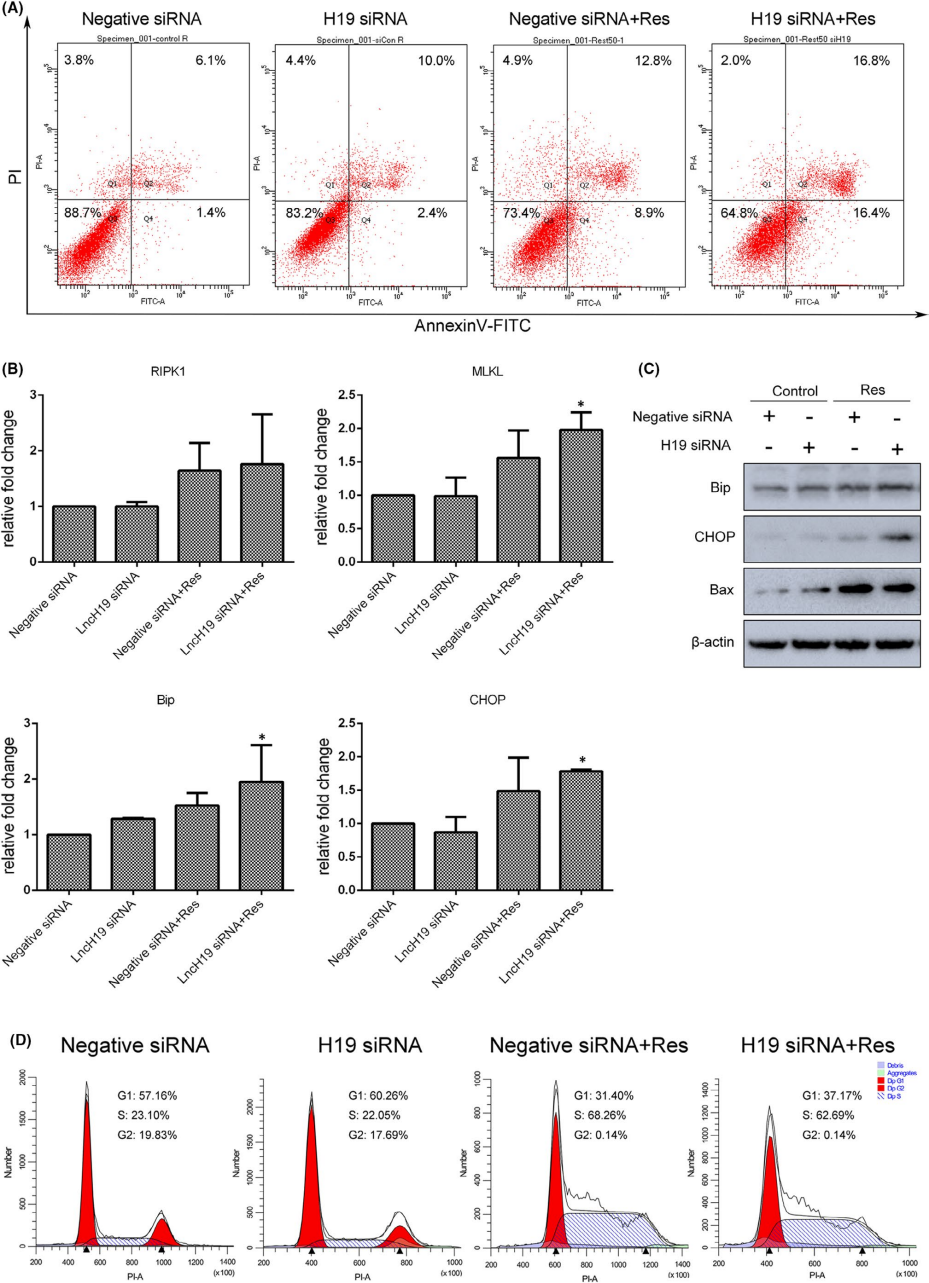
Resveratrol (Res) treatment combined with lncRNA H19 knockdown further inhibited SGC7901 cell survival. (A) Annexin V/PI staining and flow cytometry were used to assess apoptosis in cells treated with 50 μM resveratrol for 24 h combined with lncRNA H19 knockdown for 72 h. (B) Expression of necrosis‐ and ER stress‐associated genes was analysed by qRT‐PCR. (C) Levels of ER stress‐ and apoptosis‐associated proteins were analysed by Western blot. (D) Determination of cell cycle progress in combination‐treated cells. Reported values are mean ± SEM. *p < 0.05 indicates significant differences compared with the control group

**FIGURE 7:**
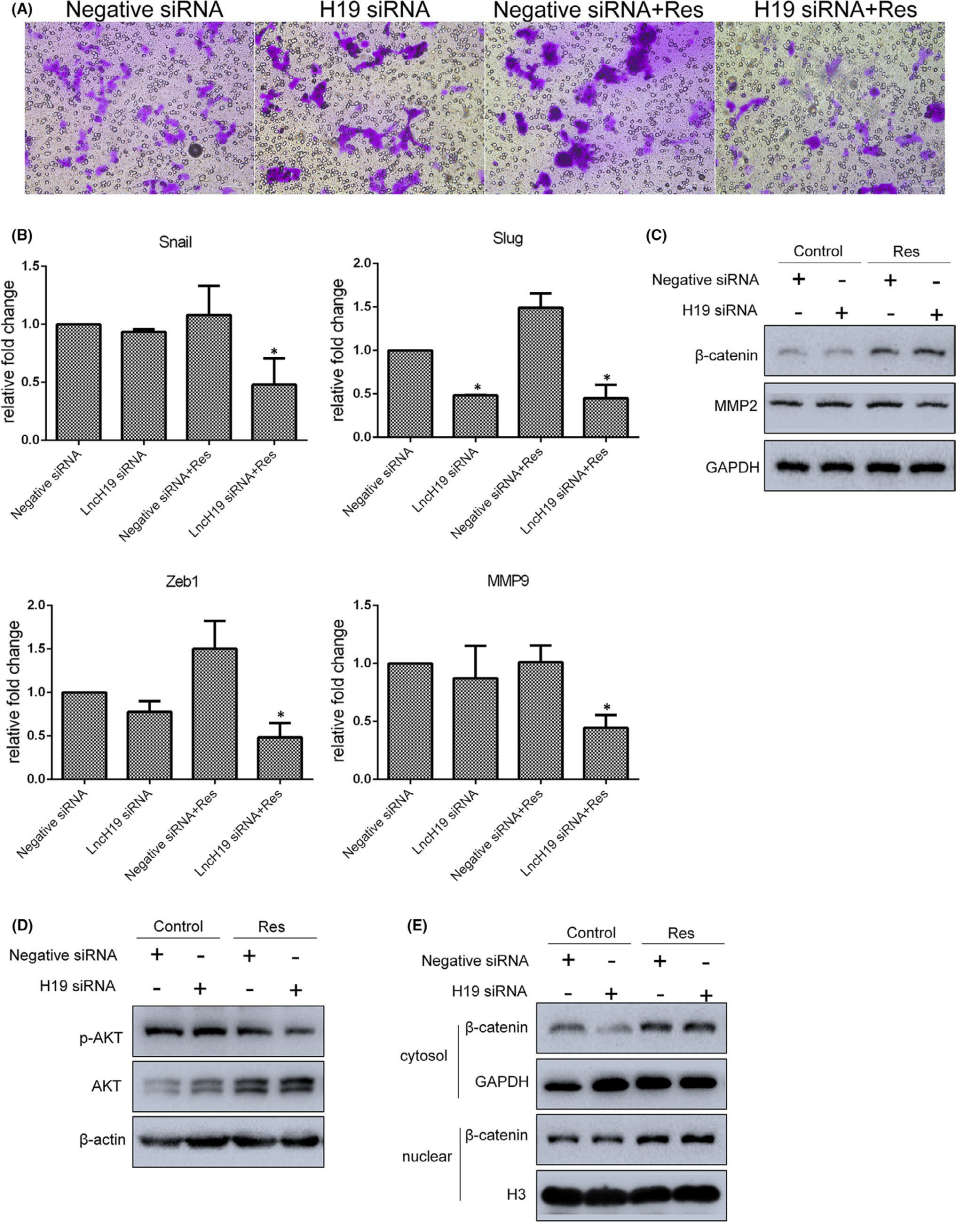
Resveratrol (Res) treatment combined with lncRNA H19 knockdown further inhibited SGC7901 cells migration and affected AKT and Wnt/β‐catenin pathways. (A) The migration of combination‐treated cells was measured by transwell assays. Cells that had undergone migration were stained with 0.1% crystal violet. (B and C) Expression of EMT‐associated genes in cells was analysed by qRT‐PCR and Western blot. (D and E) Levels of phosphorylated AKT and nuclear β‐catenin were determined by Western blot. Reported values are mean ± SEM. *p < 0.05 indicates significant differences compared with the control group

Furthermore, to confirm the function of H19 in combination with low concentration resveratrol, SGC7901 cells were subjected to a series of apoptosis, cell cycle and migration assays. As revealed by Annexin V‐FITC/PI staining, compared with the control group, the apoptosis ratios of H19 siRNA knockdown, resveratrol‐treated and combined groups were 12.4%, 21.7% and 33.2% respectively. The viability of cells treated with combined resveratrol and H19 siRNA was significantly inhibited (Figure 6A), while the expression of necrosis‐associated genes (RIPK1 and MLKL) and ER stress‐related genes (Bip and CHOP) was upregulated in these cells (Figure 6B). Consistent with these results, levels of Bip, CHOP and Bax proteins were also enhanced by the combined treatment (Figure 6C). Apoptosis and cell cycle arrest are inter‐related; flow cytometry was therefore used to evaluate cell cycle arrest in H19 siRNA knockdown and/or 50 µM resveratrol‐treated SGC7901 cells. As shown in Figure 6D, exposed to either 50 µM resveratrol or the combined treatment increased the proportion of cells in S‐phase, compared with controls. In transwell assay, H19 knockdown alone or combined treatment significantly inhibited cell migration (Figure 7A). Furthermore, expression of the metastasis‐related gene snail, slug, zeb1 and MMP9 was decreased in SGC7901 cells treated with combined resveratrol and H19 siRNAs (Figure 7B), while the levels of β‐catenin and MMP2 proteins were found to be up‐ and downregulated respectively (Figure 7C); these changes were consistent with the observed reduction in migration ability. In addition, phosphorylation of AKT was decreased in cells exposed to the combined treatment (Figure 7D). However, there was virtually no increase in β‐catenin nuclear translocate induced by 50 µM resveratrol alone or in combination with H19 knockdown, when compared to the control (Figure 7E). Overall, the role played by the Wnt/β‐catenin pathway in SGC7901 gastric cancer cells should be further determined.

## 4 DISCUSSION

There are still large numbers of natural compounds that are far from being applied in a clinical setting. Demonstration of an ability to regulate genetic and epigenetic factors would strengthen the case for using such compounds in cancer therapy.^11^, ^22^ Resveratrol has potent anti‐inflammatory, anti‐oxidative and anti‐apoptotic properties; therefore, there have been numerous studies to investigate its anti‐tumour function in recent decades. Non‐coding RNAs are a critical component of epigenetics and closely associated with tumour chemotherapy.^11^, ^23^, ^24^ In the present study, the expression of lncRNA MEG3, PTTG3P, GAS5, BISPR, MALAT1 and H19 was shown to be altered in SGC7901 cells treated with resveratrol. Of these, H19 was initially recognized as an oncofetal transcript, and aberrant increase in its expression has been observed in malignant tissues in several types of cancers.^25^ However, controversy remains regarding the potentially tumour suppressive or oncogenic role of H19. The functional role of this lncRNA may depend on cancer type, progression stage, molecular background and microenvironment.^26^ Results from the present study showed that H19 was over‐expressed at low concentration of resveratrol and downregulated at high concentrations. Silencing of H19 has been reported to prevent cell proliferation and migration in lung cancer by reducing methylation of E‐cadherin promoter.^27^ We therefore wanted to examine the impact of downregulated H19 expression on the anti‐tumour effects of resveratrol. In fact, we have previously shown that knockdown of H19 contributes to the increased sensitivity of cancer cells to pterostilbene (a dimethyl ether analog of resveratrol), reducing cell proliferation and invasiveness.^23^

Gene set enrichment analysis based on RNA‐seq found that several cell death‐related pathways were activated by resveratrol treatment, including apoptosis and autophagy. Cell death induction has become a popular treatment strategy for cancer.^7^ Resistance to apoptosis is one of the hallmarks of cancer, resulting in malignant cells that do not die.^28^ Thus, great efforts have been made to promote apoptosis in many chemotherapy strategies. This study showed that resveratrol treatment increased the rate of apoptosis in gastric cancer cells at a dose‐dependent manner. Studies have shown that silencing of H19 induced apoptosis through modulation of a variety of signalling mechanisms.^29^, ^30^ It was therefore possible that downregulation of H19 would further enhance the pro‐apoptotic effects of resveratrol treatment. As expected, resveratrol treatment in combination with H19 knockdown significantly upregulated the rate of apoptosis when compared with the control (33.2% vs. 7.5%, *p* < 0.001). Autophagic cell death is another important cell death mechanism involved in tumorigenesis and cancer therapy. Results of the present study revealed that the expression of LC3‐II was increased by resveratrol treatment in a dose‐dependent manner, suggesting activation of autophagy by resveratrol in gastric cancer cells. AKT and mTOR pathway are two vital signalling proteins involved in the normal autophagy induction^31^, ^32^; phosphorylation of both AKT and mTOR was decreased in cells after resveratrol treatment, further confirming that autophagy was activated. In addition to autophagy, abnormal activation of PI3K/AKT/mTOR signalling has also been found to regulate apoptosis, chemoresistance, EMT and metastasis in various types of human cancers.^32^ In fact, resveratrol also weakened cell migration in a dose‐dependent manner; this was potentially related to the aberrant expression of EMT‐related genes and matrix metalloproteinases. Decreased expression of E‐cadherin, ZO‐1 and β‐catenin, apparently inconsistent with the process of EMT reversal, was possibly due to the destruction of cell membranes undergoing cell death.

Endoplasmic reticulum stress can be caused by the accumulation of misfolded proteins in the ER and triggering of the unfolded protein response, which in turn induces processes that lead to the recovery of cellular homeostasis.^33^, ^34^ Various natural compounds and their derivatives have been shown to exert anti‐cancer effects by modulation of ER stress.^12^ Thus, ER stress and its constituent elements are attractive drug targets for chemotherapy. In this study, resveratrol promoted the expression of Bip and CHOP at a dose‐dependent manner, indicating that ER stress was induced. Persistent or aggravated ER stress is known to switch cancer cells from pro‐survival to pro‐apoptotic states.^12^, ^35^ Applying ER stress activators tunicamycin or thapsigargin further exacerbated the resveratrol‐induced reduction of survival in cancer cells. Autophagy is also a critical protective mechanism during ER stress, relieving stress and triggering cell death under extreme conditions, although these mechanisms can also function independently.^34^ Resveratrol combined with tunicamycin or thapsigargin further increased the expression of LC3‐II compared with resveratrol alone, while levels of MMP2 and MMP9 were also further reduced in these cells. In addition, levels of Bip and CHOP proteins were further increased in resveratrol‐treated cells after knockdown of H19. Increased ER stress therefore plays a potential role in enhancing the anti‐tumour effects of resveratrol treatment.

The Wnt/β‐catenin pathway is a conserved signalling pathway controlling diverse physiological and pathological processes, including proliferation, apoptosis, differentiation, migration and invasion.^36^ Aberrant activation of transcription factor β‐catenin, the central component of this signalling pathway, contributes to early events in tumorigenesis.^36^, ^37^ In this study, nuclear translocation of β‐catenin was decreased when cells were treated with a high concentration of resveratrol. Meanwhile, decreased levels of β‐catenin were also seen in cells treated with resveratrol and tunicamycin/thapsigargin, indicating that resveratrol‐induced downregulation of β‐catenin was closely connected with the level of ER stress. In fact, many reports have documented that β‐catenin expression is inhibited by ER stress.^38^, ^39^ Therefore, one of the reasons for the similar level of β‐catenin in cells treated with resveratrol alone or in combination with H19 knockdown was possibly due to the level of ER stress being insufficiently high.

In summary, the present study further investigated the roles of ER stress modulation and lncRNA H19 expression in resveratrol treatment, while our results indicated that regulation of ER stress and lncRNA expression may enhance the anti‐tumour function of resveratrol.

## CONFLICT OF INTEREST

The authors confirm that there are no conflicts of interest.

## AUTHOR CONTRIBUTIONS

**Tianye Li:** Formal analysis (equal); Investigation (equal); Writing – original draft (equal). **Xinyue Zhang:** Investigation (equal). **Linglin Cheng:** Investigation (equal). **Chunting Li:** Investigation (equal). **Zihan Wu:** Investigation (equal). **Yingqi Luo:** Investigation (equal). **Kunpeng Zhou:** Investigation (equal). **Yanlin Li:** Investigation (equal). **Qi Zhao:** Formal analysis (equal); Methodology (equal); Writing – review & editing (equal). **Yongye Huang:** Conceptualization (equal); Funding acquisition (equal); Project administration (equal); Supervision (equal); Writing – original draft (equal); Writing – review & editing (equal).

## Supporting information

Supplementary Material

## Data Availability

Data used to support the findings of this study are available from the corresponding author upon request.
