# Supplementary material for: Modulation of lncRNA H19 enhances resveratrol‐inhibited cancer cell proliferation and migration by regulating endoplasmic reticulum stress
Source: J Cell Mol Med. 2022 Feb 14;26(8):2205–17. doi: 10.1111/jcmm.17242 (PMC8995452; doi:10.1111/jcmm.17242)

Supplemental Materials

Modulation of lncRNA H19 enhances resveratrol-inhibited cancer cell proliferation and migration by regulating endoplasmic reticulum stress

Running Title: H19 in resveratrol treatment

Tianye Li [1], Xinyue Zhang [1], Linglin Cheng[1], Chunting Li[1], Zihan Wu [1], Yingqi Luo [1], Kunpeng Zhou [1], Yanlin Li [1], Qi Zhao [2,*], Yongye Huang [1,*]

[1] College of Life and Health Sciences, Northeastern University, Shenyang, 110169, China;
[2] School of Computer Science and Software Engineering, University of Science and Technology Liaoning, Anshan, 114051, China.

[*] To whom correspondence should be addressed: Tel: +86-24-83656116. Fax: +86-24-83656116. E-mail: zhaoqi@lnu.edu.cn (Qi Zhao), huangyongye88@163.com (Yongye Huang).

1.Spplemental procedure

1.1 Cell Culture

Human gastric cancer cells SGC7901 and BGC823 and lung cancer cells A549 were cultured in DMEM medium or RPMI 1640 medium supplemented with 10% fetal bovine serum, 1% non-essential amino acid, 1% glutamine, 100 U/mL penicillin, and 100 mg/mL streptomycin at 37°C in humidified incubator with 5% CO_2. Cells were routinely passaged at 70%~80% confluent density using a 0.25% trypsin solution.

1.2 siRNA Transfection

To knockdown lncRNA H19 in SGC7901 cells, siRNA 5′ - CCCACAACAUGAAAGAAACTT -3′ and 5′ - GCUAGAGGAACCAGACCUUTT -3′ were used in this study. As a control for these studies, a non-silencing negative siRNA sequence obtained from Genecreate (Wuhan, China) was used. The siRNA was transfected into cells using Lipofectamine 3000 reagent (Invitrogen, Shanghai, China) according to the manufacture's guidelines. A final concentration of the siRNA was 100 nM. After being incubated with lncRNA H19 siRNA for 24 hours, cells were re-transfected for another 48 hours. Resveratrol was added to the cells when lncRNA H19 knockdown for 48 hours and treated SGC7901 cells for another 24 hours. Finally, the knockdown cells were harvested after 72 hours.

1.3 Drugs

Resveratrol was purchased from Sigma-Aldrich. The resveratrol powder was dissolved in DMSO into 50 mM stock solution and stored at -20°C condition, then diluted in culture media prior to experimentation. The complete medium DMEM with the same volume of DMSO was used as the control.

1.4 Cell Cycle and Apoptosis Assay

Cell cycle distribution and apoptosis were examined via a flow cytometer (Fortessa, BD Biosciences, New York, USA), and the results were analyzed by ModFit software. 6×10^5 cells were seeded in the 6 cm dishes and harvest after 24 h with various concentration resveratrol treatment or 72 h with lncRNA H19 siRNA transfection.

For cell cycle analysis, cell pellets were washed with cold PBS and fixed in 70% ice-cold ethanol in -20°C overnight. The fixed cells were stained in PI/RNase Staining Buffer Solution (BD Biosciences,

New York, USA) for 30 min at room temperature in the dark according to the manufacturer's instruction. An Annexin V/PI double staining apoptosis detection kit was used to detect cell apoptosis. After resveratrol and/or lncRNA H19 knockdown treatment, the adherent and suspended cells were harvest and washed with cold PBS. Then cells were incubated in 100 μL 1×binding buffer containing Annexin V-FITC and PI at room temperature for 15 min in the dark according to the manufacturer's instruction. Thereafter, 400 μL 1×binding buffer was added and the flow cytometer as mentioned above was utilized to detect the apoptosis.

1.5 Quantitative Real-Time PCR

qRT-PCR was used to detect the mRNA expression in treated SGC7901 cells. In brief, the TRIzol reagent (Tiangen Biotech, Beijing, China) was used to isolate the total RNA and the All-in-One cDNA synthesis SuperMix (Bimake, Shanghai, China) was used to reverse-transcribed 2 μg total RNA into cDNA according to the manufacturer's protocol. Then, the 2×SYBR Green qPCR Master Mix (Bimake, Shanghai, China) was used to detect the mRNA expression following the manufacturer's protocol on a CFX96 real-time PCR detection system and GAPDH was used as a reference gene. The relative expression of the target genes was calculated using the $2^{-\Delta\Delta Ct}$ method.

2. Supplemental table

Supplemental Table 1. Primer sequences for qRT-PCR

Genes	Forward	Reverse
ATG3	GCCGTTAAAGAGATCACACTGG	CATAGCCAAACAACCATAATCGTGG
ATG5	CAGCTCTTCCTTGGAACATC	GGCTGTGGGATGATACTAATATG
Beclin1	GAAGACGTGGAAAAGAACCGC	CAGCCTGAAGTTATTGATTGTGC
β-catenin	GATTTGATGGAGTTGGACATGG	TGTTCTTGAGTGAAGGACTGAG
Wnt3a	TGCATAGGCTCCTTCCTGTGG	TGGCTGGTGGGCTGAATTTC
Bip	ACGTGGAATGACCCGTCTGT	AACCACCTTGAACGGCAAGA
CHOP	CAGAGCTGGAACCTGAGGAG	TGGATCAGTCTGGAAAAGCA
E-cadherin	GGATTGCAAATTCCTGCCATTC	AACGTTGTCCCGGGTGTCA
N-cadherin	GACGGTTCGCCATCCAGAC	TCGATTGGTTTGACCACGG
Fibronectin	TGACCTTTTCTGGCTCGTCT	GTTCAGCACAAAGGGCTCTC
Snail	GCTGCAGGACTCTAATCCAGAGTT	GACAGAGTCCCAGATGAGCATTG
MMP9	TTGGTCCACCTGGTTCAACT	ACGACGTCTTCCAGTACCGA
RIPK1	GCACCGCTAAGAAGAATGG	GCCACACAATCAAGTTGAAGAG
MLKL	GCTGAGTGATGTCTGGAAGG	CTTTGGAATCGTCCTCTGGG
Slug	GCGATGCCCAGTCTAGAAAA	GCAGTGAGGGCAAGAAAAAG
Zeb1	GCCAATAAGCAAACGATTCTG	TTTGGCTGGATCACTTTCAAG
GAS5	TGGTTCTGCTCCTGGTAACG	AGGATAACAGGTCTGCCTGC
MEG3	CTGCCCATCTACACCTCACG	CTCTCCGCCGTCTGCGCTAGGGGCT
MALAT1	GACGAGTTGTGCTGCTATCTT	GATTCTGTGTTATGCCTGGTTAG
BISPR	GCCAACAAACAATGTCGGTCT	CAGAGACACAGATGCTGCCTAA
DICER1-AS1	TGACCAGTCTTACCCCTCCT	CTGAAGCACCTGAAATGCG
LINC01121	GGAAGAAGTGTGCTGTGCCA	CAACCCCTGACTCCTACACG
TUG1	CTGAAGAAAGGCAACATC	GTAGGCTACTACAGGATTTG
PTTG3P	GGGGTCTGGACCTTCAATCAA	GCTTTAGGTAAGGATGTGGGA
H19	AAGAAGGAGGTTTAGGGGATCG	CCGAGAAGATGTCACCTTTGCT
GAPDH	GACAGTCAGCCGCATCTTCT	TTAAAAGCAGCCCTGGTGAC

3. Supplemental figures

Figure S1 Expression of lncRNAs in resveratrol-treated cells and viability of other cancer cell lines exposed to resveratrol. (**A, B**) Cell survival rate and expression of lncRNA H19 in A549 and BGC823 cells treated with 0, 50, 100 and 200 μM resveratrol for 24 h. Expression of H19 was determined by qRT-PCR. (**C**) SGC7901 cells were treated with resveratrol at different concentrations (0, 50, 100 and 200 μM) for 24 h and gene expression was determined by qRT-PCR. Reported values are mean ± SEM. *$p < 0.05$, **$p < 0.01$ and ***$p < 0.001$ indicate significant differences compared with the control group.

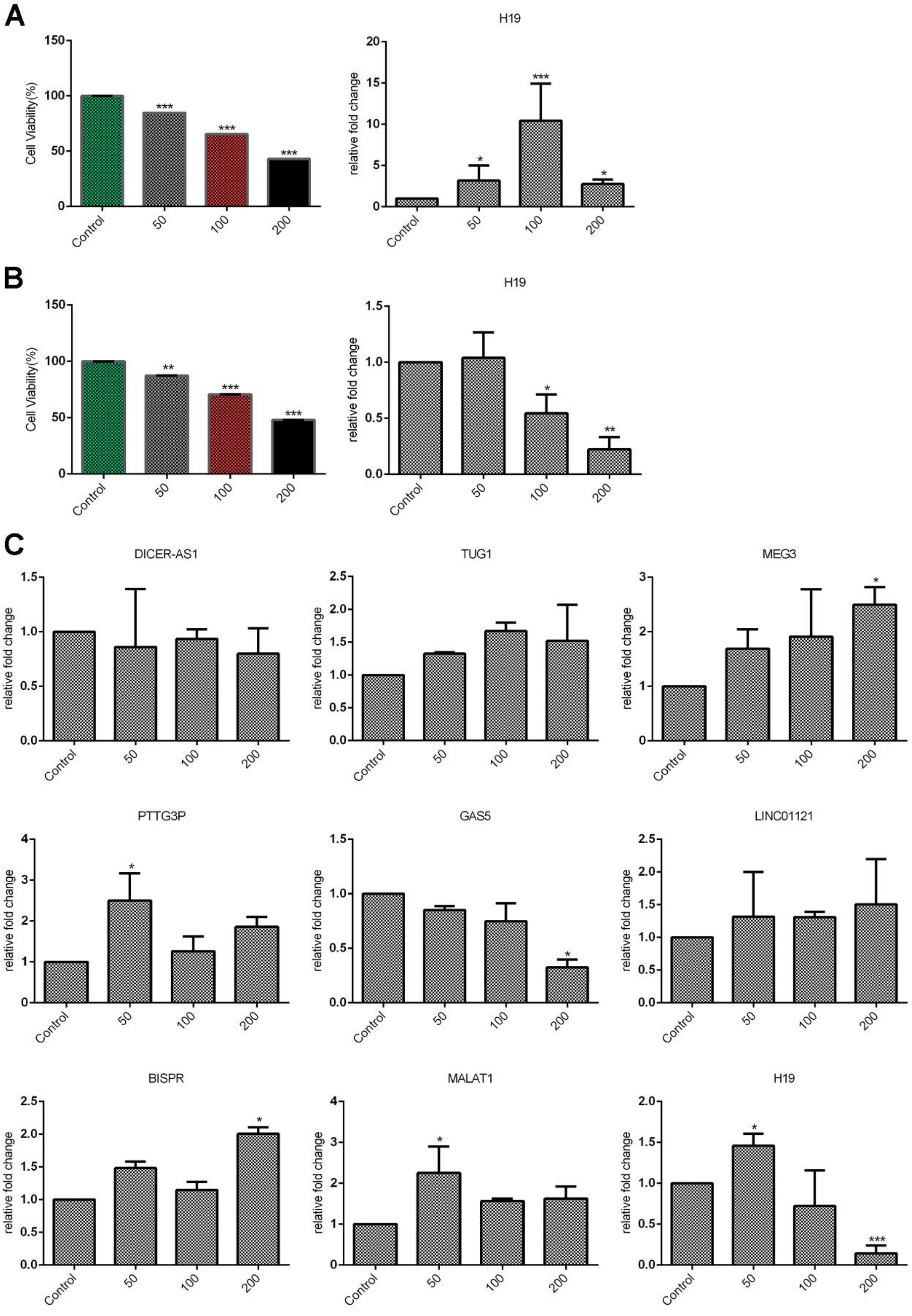
A
Cell Viability(%)
150
100
50
0
***
***
***
Control
50
100
200
H19
relative fold change
20
15
10
5
0
*
***
*
B
Cell Viability(%)
**
***
***
H19
relative fold change
1.5
1.0
0.5
0.0
*
**
C
DICER-AS1
TUG1
MEG3
PTTG3P
GAS5
LINC01121
BISPR
MALAT1
H19
relative fold change

Figure S2 Results of RNA-seq analysis in resveratrol-treated A549 lung cancer cells (50 μM for 24 h). (**A**) GO analysis of significant upregulated genes. (**B**) KEGG pathway analysis of significant upregulated genes. (**C**) Heatmap of any changed lncRNAs.

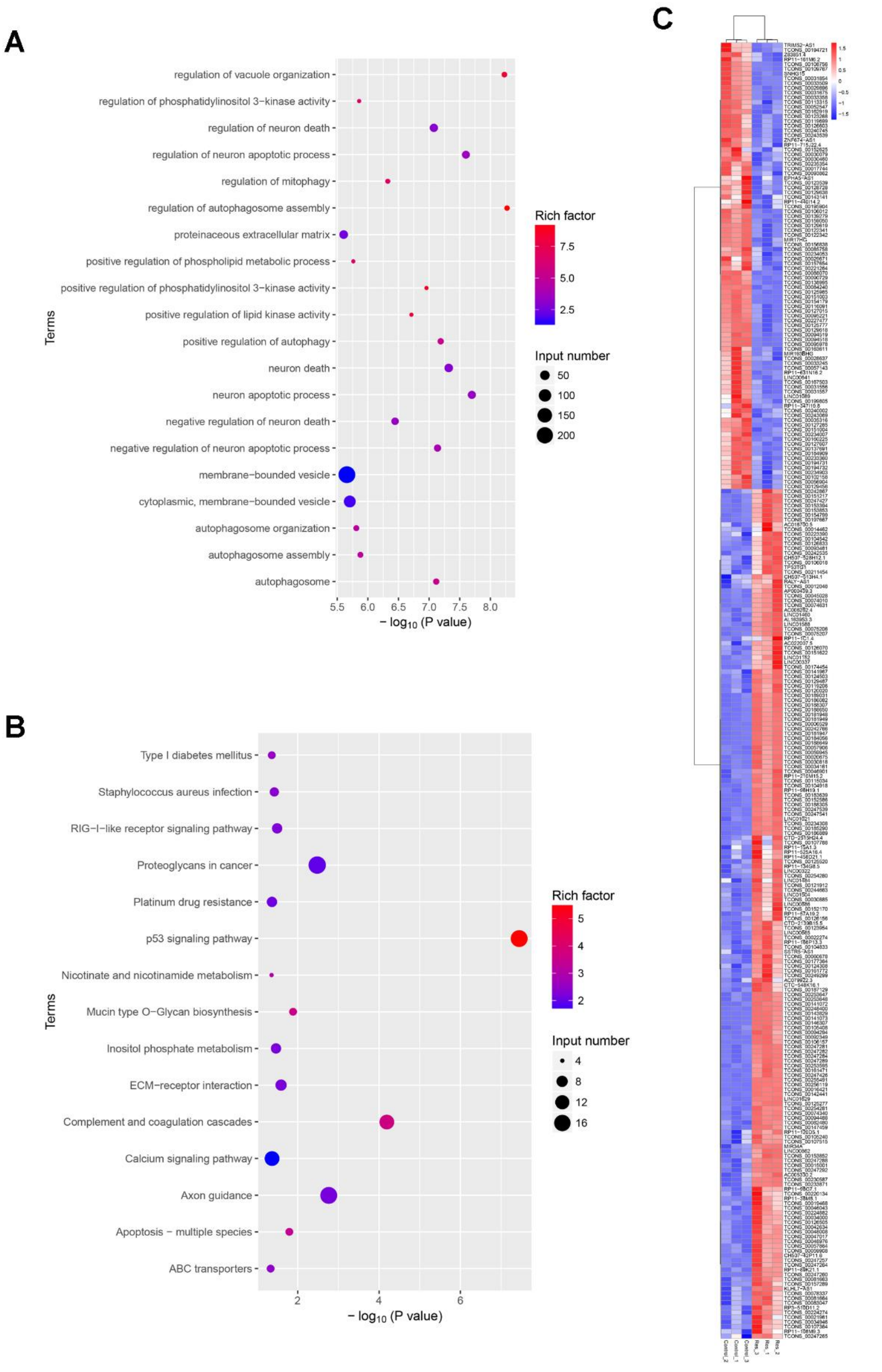

Figure S3 GSEA analysis was performed on RNA-seq data. p53-related pathways were upregulated in resveratrol-treated cells. Myc-associated pathways were downregulated in resveratrol-treated cells.

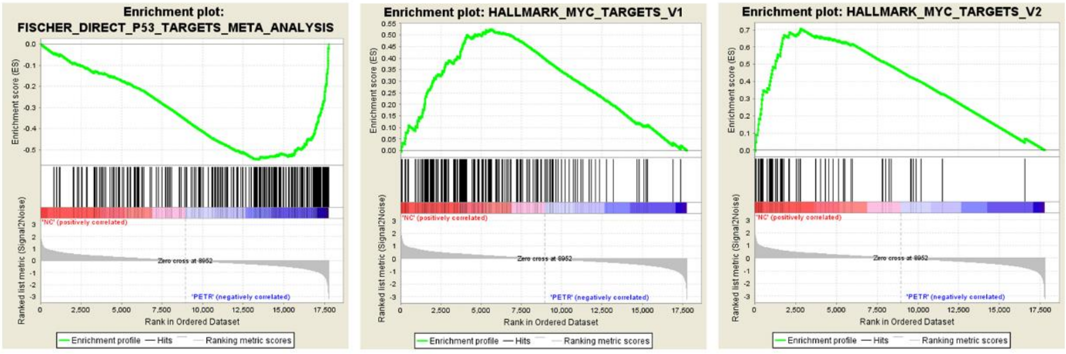

Figure S4 Expression of lncRNAs in resveratrol (Res)- and H19 siRNA-treated SGC7901 cancer cells. SGC7901 cells were treated with H19 siRNA for 72 h and 50 μM resveratrol for 24 h, with gene expression determined by qRT-PCR. Reported values are mean ± SEM. *p < 0.05, **p <0.01 and ***p <0.001 indicate significant differences compared with the control group.

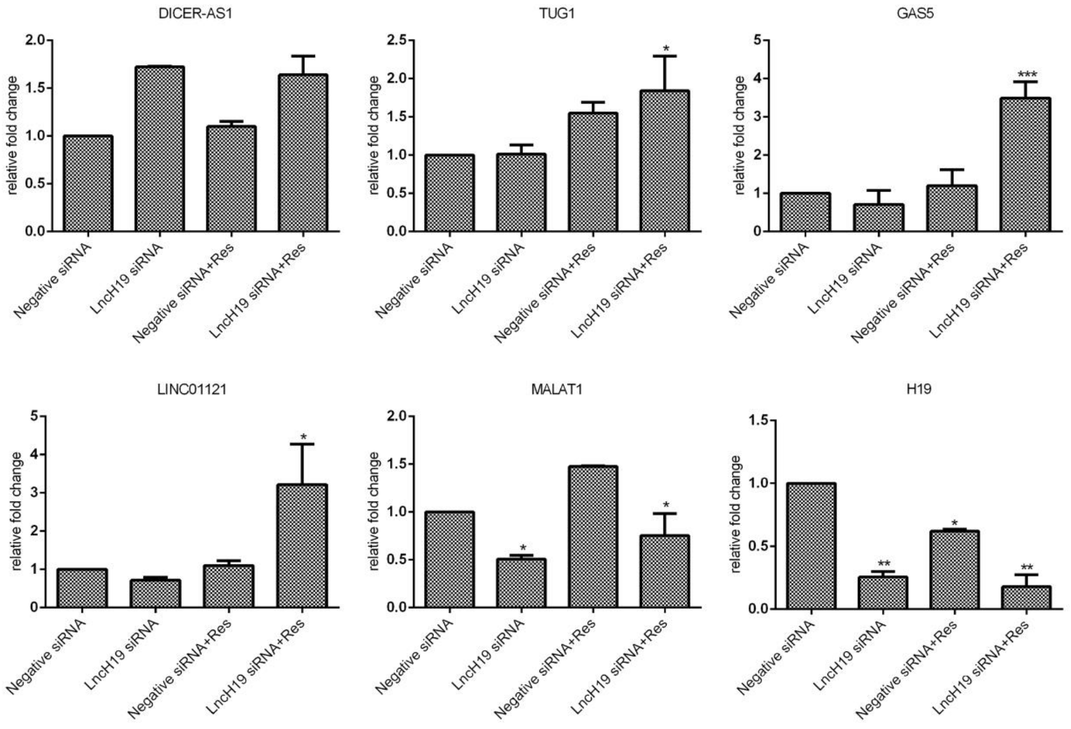

Supplement: Supplementary file 1 — Supplementary Material [file JCMM-26-2205-s001.pdf]
